# Mercury bio-extraction by fungus *Coprinus comatus*: a possible bioindicator and mycoremediator of polluted soils?

**DOI:** 10.1007/s11356-015-5971-8

**Published:** 2015-12-26

**Authors:** Jerzy Falandysz

**Affiliations:** Laboratory of Environmental Chemistry and Ecotoxicology, Gdańsk University, 63 Wita Stwosza Str., PL 80-308 Gdańsk, Poland

**Keywords:** Foraging, Fungi, Mycoremediation, Shaggy Ink Cap

## Abstract

The Shaggy Ink Cap (*Coprinus comatus*), which is a common in wild in northern hemisphere was examined in field for potential to be used as possible bio-extractor of Hg from polluted grounds but also as possible bioindicator of urban soils (roadside, barren lands, lawns) pollution with Hg. The contents of Hg in caps and stipes of *C. comatus* from the grounds examined in this study correlated positively with the levels of soil contamination. Analysis of sets of data available worldwide on Hg in *C. comatus* and soils beneath-fruiting bodies showed on a positive correlation between degree of soil and mushroom contamination. Hence, *C. comatus* could be considered as a sensitive species and with bioindication and bioremediation potency for soils polluted with Hg in further studies. Young-fruiting bodies of *C. comatus* are edible and considered excellent if consumed soon after pick-up. Eating them when foraged from the urban places can provide to a consumer Hg at relatively high dose, while unresolved question is absorption rate of Hg compounds contained in ingested mushroom meal.

## Introduction

Environmental pollution with heavy metals such as Cd, Hg, Pb, and their accumulation in soils due to the industrial activities, urbanization, and traffic is an ongoing process, and various efforts are undertaken to effectively reduce and eliminate the sources as well as to restore degraded grounds (McGrath and Zhao 2003; Xu et al. 2015). One of the biological techniques of soil restoring discussed in scientific literature is mycoremediation using macrofungi (Gadd et al. 2012).

Macrofungi are well known for their ability to efficiently absorb various metallic elements and metalloids from the substrata and to sequester them in their fruiting bodies (Byrne and Tušek-Žnidarič 1990). Hence, fruiting bodies of edible and inedible mushrooms can be relatively rich in inorganic constituents, and data published on metals and minerals composition and content of macrofungi is much more when compared to that on their potential to bio-extract elements (Falandysz and Borovička 2013; Falandysz et al. 2001b; Tel et al. 2014).

For example, *Agaricus campestris* is good for sequestering Ag (Falandysz and Danisiewicz 1995); *Agaricus macrosporus* for Cd (Garcíá et al. 2005); *Amanita muscaria* for Cd, Cu, V, Hg (Lepp et al. 1987; Drewnowska et al. 2013); *Boletus edulis* for Cd, Cu, Hg (Falandysz et al. 2011; Frankowska et al. 2010); *Macrolepiota procera* for Ag, Cu, Cd, Hg (Gucia et al. 2012a and 2012b); *Laccaria amethistina* and *Laccaria vinaceoavellanea* for As (Zhang et al. 2015); *Paxillus involutus* for Cu, Zn (Brzostowski et al. 2011a, and 2011b); *Suillus grevillei* for Ag, Cd, Hg (Chudzyński and Falandysz 2008; Chudzyński et al. 2009); *Xerocomus badius* for Ag, Cd, Hg (Falandysz et al. 2012; Kojta et al. 2012; Mleczek et al. 2015); *Xerocomus subtomentosus* for Ag, Cd, Hg, (Chojnacka et al. 2012; Jarzyńska et al. 2012). Some mushrooms have also capacity to hyperaccumulate certain elements in flesh, e.g., *Amanita strobiliformis* and *Amanita submembranacea* for Ag (Borovička et al. 2007, 2010).

Fungi in a few original studies has been postulated or tested as biota more or less useful in process of removing or decreasing the content of the hazardous metallic elements from degraded soils or contaminated compost, e.g. with Hg and *Pleurotus ostreatus* (Bressa et al. 1998), Ag and *Agaricus bisporus* (Byrne and Tušek-Žnidarič 1990; Falandysz et al. 1994), Cd, Cu, Hg, Pb and *Armillaria mellea*, *Polyporus squamosus*, *Polyporus suiphureus* (Demirbaş 2002); Ca, Cs, K, Na and *Pleurotus eryngii* (Bystrzejewska-Piotrowska et al. 2008), Cu by *Oudemansiella radicata* (Jiang et al. 2015), ^239^Pu and ^241^Am and *P. ostreatus* (Galanda et al. 2014), Ag, Pb, Th, U and several species of mushrooms for which accumulation of those metals in fruiting bodies apparently does not depend on total content and chemical fractionation of these metals in soils (Kubrová et al. 2014).

Nevertheless, practical solutions are lacking and fishing for most suitable species continues. Recently, it has been shown in a pot study that *Coprinus comatus* in presence of chelating agents such as ethylenediaminetetraacetic acid (EDTA) or nitrilotriacetate (NTA) very efficiently take-ups of Cd, Cu, and Pb from soil (Cen et al. 2012), and similar study with added chelators has been conducted for *Tricholoma lobayense* Heim (Wang et al. 2012). A potential for bio-extraction of Pb from low-polluted soils has been shown for *Oudemansiella radicata* (Zhang et al. 2012).

Mercury is a particular example of environmental and food toxicant because of high toxicity and biomagnification of methylmercury (MeHg) in food chains. Mercury, because is highly volatile could be emitted due to any high temperature process (e.g., combustion of biomass or waste, production of cement, metal ore refining). At the local or regional scale, soil can become a hot spot polluted with Hg because of fumes from the nonferrous metals producing facilities, use of the Hg compounds as the catalyst in an organic chemicals manufacturing, or an improper storage and disposal of the Hg containing materials, products, and wastes (Hu and Cheng 2012). Hence, both identifications of current and forgotten places polluted with this element as well as development of remediation techniques are the actual needs.

As mentioned, an exceptional feature of mushrooms when compared to vascular plants is efficient accumulation by them of mercury (Cibulka et al. 1996), and some examples are available on bioconcentration of Hg by several species both the ectomycorrhizal and saprophytic mushrooms (Falandysz 2002; Falandysz et al. 2001a, 2002a, b, 2003a, b, c and 2014b; Krasińska and Falandysz 2015a, b; Melgar et al. 2009; Nasr and Arp 2011; Rieder et al. 2011). The rates of transfer (bioconcentration) of Hg sequestered in fruiting bodies by mushrooms depend on species and decrease with increasing soil/substratum Hg content because of limited natural capacity for accumulation (Bargagli and Baldi 1984; Falandysz et al. 2012; Falandysz and Drewnowska 2015a), while some species will tolerate highly polluted grounds because of cinnabar mining and can accumulate Hg at remarkably great concentration (Árvay et al. 2014). Also, mushrooms with deeper mycelia when emerged from red and yellow lateritic soils enriched with Hg in the mineral layer because of occurrence of the Circum-Pacific Mercuriferous Belt could accumulate Hg in fruiting bodies at highly elevated concentration (Falandysz et al. 2015a, b; Kojta et al. 2015; Wiejak et al. 2014).

Nevertheless, this is difficult to find in nature a such good example positive relationship, at local scale but exceptions probably happen if there is diversity of Hg concentration in soil substrate to a given species of mushroom. For example, when studding mercury (Hg)—total Hg for some mushrooms and places, a good correlation has been found between soil (substrata) Hg and mushroom Hg for *Macrolepiota procera* (Falandysz and Chwir 1997). The same was for methylmercury (MeHg) and a set of four samples (three individuals of *Boletus* (*Xerocomus*) *badius* - current name *Imleria badia* and one of *Leccinum scabrum*) form abandoned Hg mine (Fischer et al. 1995) but this was not exactly the same for inorganic Hg.

A layer(s) in soil where mycelia lives and layer(s) in soil where Hg is contained and available matter. Deposition of airborne mercury from a long-range transport in pristine region of the Himalayan size Mountain Gongga (Minya Konka) in the eastern Tibetan plateau is the only explanation for elevated Hg content in the *Gymnopus erythropus* and *Marasmius dryophilus*, which both have shallow mycelia (Falandysz et al. 2014a). In a case of mushrooms that have deeper mycelia a diversification of Hg contents, as observed for several species of *Leccinum* and *Boletus* mushrooms from the Yunnan Province in China, could rather reflect the local/regional differences of Hg in the mineral layer of soil due to the geochemical anomalies but not because of fallout from the anthropogenic emissions from the beginning of the era of industrialization of the world (Falandysz et al. 2015a, b).

*Coprinus comatus* (O.F. Müll.) Pers. that is commonly named as Shaggy Ink Cap, Shaggy Mane, Shaggy Parasol, or Lawyer's Wing and is popular saprophytic mushroom in moderate climate, and its young-fruiting bodies are edible (Lassoe et al. 1996). This mushroom can be found emerging from the ground on the city lawns, abandoned grounds, parks, along the roads, and waste areas, and hence is rather rare example of edible wild mushroom that survives unhostile urban condition and can be picked-up both in the cities and in villager areas. This species is picked up by some consumers even if emerged at grounds in populated city. The *C. comatu*s is also cultivated in China as food.

Aim of this study was to examine, if the Shaggy Ink Cap, which is a common in wild in northern hemisphere, has any potential to be used as possible bio-extractor of Hg from polluted grounds but also as possible bioindicator of urban soils (roadside, barren lands, lawns) pollution with Hg. Also, estimated was the possible intake rate of total mercury by consumers of *C. comatus* at the region investigated. Data available on Hg accumulated in *C. comatus* from Europe and Asia were summarized.

## Materials and methods

**Fig. 1:**
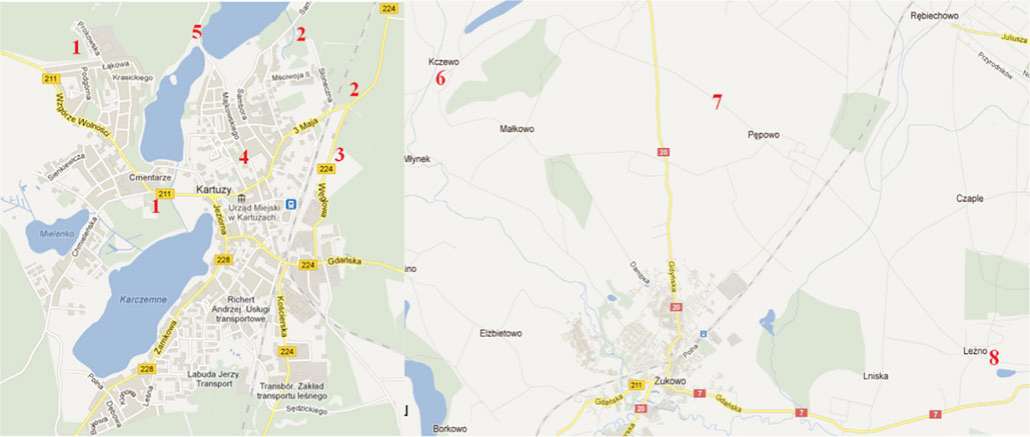
Localization of the sampling places of *C. comatus* and soils in the town of Kartuzy (54°20'06" N and 18°12'05" E; 1–5) and elsewhere: Kczewo (54°23'00" N and 18°20'00" E; 6), Pępowo (54°38'66" N and 18°40'23" E; 7) and Leźno (54°21'0" N and 18°26'0" E; 8) in Pomerania land in northern part of Poland (Google maps; color figure available in online version)

The specimens of fruiting bodies of *C. comatus* and topsoil (0–10-cm layer) samples beneath to them were collected at several sites from an area of the town of Kartuzy (14866 people) in Kaszuby region of the Pomerania land in the northern part of Poland in 2011 (Fig. 1; Table 1). Two sampling places assigned respectively with number 1, and two other with number 2 (Fig. 1) were considered as of the similar character. Hence, mushrooms and soil samples collected at two places with number 1 were integrated to make composite samples, and the same was in the case of material collected at the places assigned with number 2. The region of Kartuzy is surrounded by farmland, woodland and lakes, while apart from tourism in summer an industrial activity is little and restricted to small wood processing facilities and processing of crops.

**Table 1:** Summary of details on materials *Coprinus comatus*

Place ID	Name of the place	Type of material	
		Fungus	Soil
1	Kartuzy, Wzgórze Wolności/ Prokowska Str.	3 (13)^b^	3
2	Kartuzy, Sambora Str./3 Maja Str.	3 (13)	3
3	Kartuzy, Węglowa Str.	3 (15)	3
4	Kartuzy, Majkowskiego Str. (center of town)	3 (13)	3
5	Kartuzy, Majkowskiego Str. (northern edge)	3 (13)	3
6	Kczewo village	3 (15)	3
7	Pępowo village	3 (13)	3
8	Leźno village	3 (13)	3

Samples and total number of fruiting bodies per place (in parentheses)

**Fig. 2:**
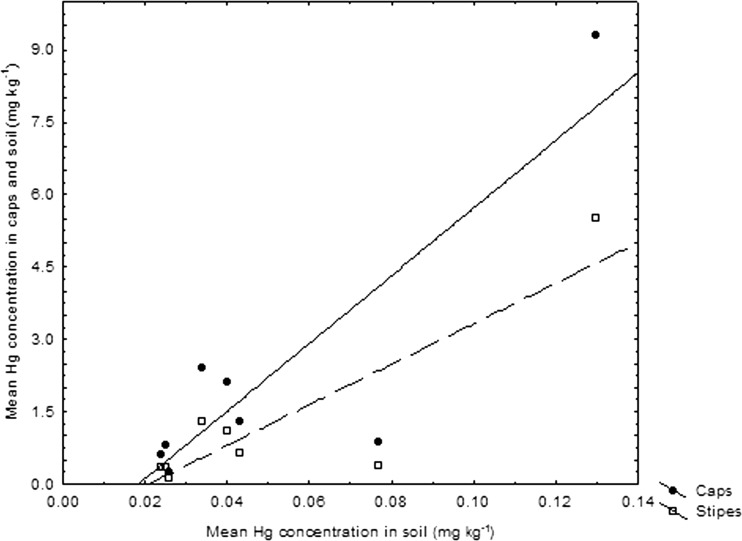
Relationships between Hg content in the caps and stipes of *Coprinus comatus* and Hg in soil substratum (for caps: y = −1.2933 + 70.1161*x; *r* = 0.87; *p* < 0.005; *r* ^2^ = 0.75; *p* = 0.0004; for stipes: y = −0.8774 + 41.9787*x; *r* = 0.87; *p* < 0.005; *r* ^2^ = 0.75) for locations examined

^a^numeration of the sampling place (see Figs 1 and 2)

^b^number of pooled

A “young” (white) of edible quality fresh-fruiting bodies, after clean up with a plastic knife from any visible plant vegetation and soil substrate debris and bottom part of the stipe was cut off, was separated into two parts—cap and stipe. Next, they were in situ sliced and dried (initially in ambient temperature for 0.5–2 h) and further were placed into plastic basket of an electrically heated commercial dryers (dehydrator for mushrooms, fruits, vegetables and herbs; model: MSG-01; MPM Product, Milanówek, Poland) and dried at 65 °C to constant mass. Dried mushrooms parts were respectively pooled and pulverized in a porcelain mortar and kept in brand new sealed polyethylene bags under dry conditions. The soils (0–10-cm layer) and litter samples free of any visible organisms, small stones, sticks, and leaves were air dried at room temperature for several weeks under clean condition. Next, the soil samples were sieved through a pore size of 2-mm plastic sieve and sealed in brand new polyethylene bags and kept under dry and clean condition. Mushrooms and soil samples were pooled, respectively (Table 1).

Mercury was determined using a direct sample matrices thermal decomposition and cold-vapor atomic absorption spectroscopy (CV-AAS; Mercury analyzer type MA-2000, Nippon Instruments Corporation, Takatsuki, Japan) (Jarzyńska and Falandysz 2011; Nnorom et al. 2013). The accuracy of the method was evaluated and further controlled by examination of the fungal-certified reference materials (CRM) and reagent blanks (both with every set of 5 fungal or soil samples). The CRM fungal material used were dried-fruiting bodies of Cow Bolete (*Suillus bovinus*; code: CS-M-1) and the Basma 5 tobacco leaves code: INCT-OBTL-5, both produced by the Institute of Nuclear Technology and Chemistry (ICHTJ), Warsaw, Poland). The content of Hg in CS-M-1 declared by the producer is 0.174 ± 0.018-mg kg^−1^ dry matter (dm), while our measurements obtained in separate trials showed 0.168 ± 0.009 mg kg^−1^ dm (*n = 5*). Declared content of Hg in INCT-OBTL-5 is 0.021 ± 0.001-mg kg^−1^ dm, and our result showed 0.020 ± 0.006-mg kg^−1^ dm (*n = 5*). For mushrooms and the soil substrates, the limit of detection was 0.005-mg kg^−1^ dm, and the quantification limit was 0.0015-mg kg^−1^ dm. One blank sample and one certified reference material sample were examined with each set of 3–10 samples studied.

Intake rate of total mercury was estimated based on median the values of mercury concentrations noted in fruiting bodies, possible intake rates of mushroom, and a provisionally tolerable intake limits of element to adult human.

The computer software Statistica, version 10.0 (Statsoft Polska, Kraków, Poland), was used for statistical analysis of data and for graphical presentation of the results of two-dimensional multiple scatter plot relationships between the variables.

## Results and discussion

The Shaggy Ink Cap seems to be a sensitive boindicator of urban soils pollution with Hg that is efficiently sequestered by this species in fruiting bodies—both caps and stipes (Table 2). The median values of BCF from the stand where topsoil showed Hg at 0.13-mg kg^−1^ dm (no 3, Fig. 1; Table 1) reached up to 73 for caps and 30 for stipes. Also, Hg content of fruiting bodies from that stand was high and median value was 9.2-mg kg^−1^ dm in caps and 5.2-mg kg^−1^ dm in stipes, An elevated content of Hg in topsoil and hence also in flesh of mushrooms at the stand no 3 could be explained by character of the ground usage there, where a scrap-heap and recycling company is active from a year. At two other places in Kartuzy (nos. 2 and 4, Fig. 1; Table 1), the median values of Hg in caps were between 2.5 and 3.0-mg kg^−1^ dm (Table 2), which are values within a range of the concentrations determined in *C. comatus* from several urban places in Europe (Table 3). At the places west (no 1) and north (no 5) of the center of the town (Fig. 1)-fruiting bodies of *C. comatus* were less contaminated, while much less were the mushrooms and soil collected in outskirts of two villages east of the town (nos. 6 and 7). At the Leźno place that is subjected for an intense road traffic, both topsoil and mushrooms showed an intermediate Hg content (Table 2). The geochemical background value of Hg (total Hg) suggested for soils of Poland is 0.05-mg kg^−1^ dm (PIG 2013), while the forest soils did contain in a top 0–10-cm layer much less of Hg than 0.05-mg kg^−1^ dm (Drewnowska et al. 2012 and 2014; Drewnowska and Falandysz 2015). In view of those figures, the soil sampled in the town of Kartuzy as well as other places (Fig. 1) can be considered as more or less contaminated with Hg from the anthropogenic emissions.

**Table 2:** Total mercury concentrations in caps and stipes of *Coprinus comatus* and beneath soil (mg kg^−1^ dm), the values of Hg caps to stipes concentration quotient (Q_C/S_), and bioconcentration factor (BCF) values

Place ID	Hg in mushrooms		Q_C/S_	Hg Soil	BCF_c_	BCF_s_
	Caps	Stipes				
1	0.88 ± 0.26	0.39 ± 0.90	1.7 ± 0.5	0.077	12 ± 3	7.5 ± 4.5
	(0.59–1.1)	(0.29–0.96)	(1.1–2.1)	(0.068–0.083)	(7.8–14)	(3.8–13)
	0.98	0.47	2.0		13	6.2
2	2.1 ± 1.1	1.1 ± 0.6	2.0 ± 0.1	0.04^a^	52 ± 27	27 ± 15
	(0.89–3.0)	(0.42–1.6)	(1.9–2.0)		(22–62)	(22–74)
	2.5	1.1	2.0		62	30
3	9.3 ± 0.5	5.5 ± 0.7	1.7 ± 0.1	0.13^a^	74 ± 4	43 ± 5
	(8.4–10)	(5.0–6.3)	(1.6–1.8)		(70–78)	(39–50)
	9.2	5.2	1.7		73	30
4	2.4 ± 1.6	1.3 ± 0.8	1.7 ± 0.5	0.034^a^	70 ± 46	38 ± 23
	(0.58–3.6)	(0.47–2.1)	(1.2–2.2)		(17–100)	(14–60)
	3.0	1.4	1.7		88	30
5	0.79 ± 0.46	0.34 ± 0.21	2.3 ± 0.2	0.025^a^	31 ± 18	14 ± 8
	(0.35–1.3)	(0.14–0.56)	(2.2–2.5)		(14–50)	(5.6–22)
	0.75	0.33	2.3		30	13
6	0.25 ± 0.02	0.12 ± 0.02	2.3 ± 0.2	0.026^a^	9.5 ± 0.8	4.8 ± 0.7
	(0.24–0.28)	(0.11–0.15)	(4.3–5.6)		(8.9–10)	(5.6–22)
	0.24	0.12	1.9		9.2	13
7	0.61 ± 0.08	0.35 ± 0.03	1.8 ± 0.4	0.024^a^	26 ± 3	15 ± 1
	(0.54–0.70)	(0.32–0.38)	(1.5–2.2)		(23–29)	(13–16)
	0.61	0.35	1.6		25	15
8	1.3 ± 0.2	0.63 ± 0.15	2.2 ± 0.2	0.043^a^	31 ± 5	15 ± 4
	(1.2–1.6)	(0.49–0.79)	(2.0–2.4)		(27–36)	(11–18)
	1.3	0.62	2.1		30	14

^a^pooled samples for soil

**Table 3:** Summary of data on total mercury concentrations in *C. comatus* and beneath soil, bioconcentration factor (BCF), and cap to stipe Hg content ration quotients (Q_C/S_)

Information	Hg mg kg dm^−1^		BCF	Reference
	Mushroom	Soil		
Norway, background	1.7/0.85 (cap/stipe)			Allen and Steinnes 1978
Finland (*n* = *2*)^#^	5.6 (1.5–9.8)			Laaksovirta and Lodenius 1979
Finland, rural (*n = 3*)	2.7 (2.5–2.9)			Kuusi et al. 1981
Finland, urban (*n = 37*)	4.7 (0.68–17)			Kuusi et al. 1981
Finland, urban (*n = 55*)	3.8 (1.4–10)			Lodenius et al. 1981
Finland, lead processing area (*n = 2*)	2.1 (0.6–3.5)			Liukkonen-Lilja et al. 1983
Finland (*n = 1*)	6.7			Kojo and Lodenius 1989
Germany (*n = 6*)	1.2 (0.4–2.2)			Seeger and Nützel 1976
Germany, Hg mining area (*n = 2*)	144	83	1.7	Fischer et al. 1995
Switzerland (*n = 1*)	2.8			Stijve and Roschnik 1974
Switzerland (*n = 4*)	3.3 (0.57–8.0)			Quinche and Dvorak 1975
Switzerland (*n = 7*)	3.1 (0.51–5.6)			Quinche 1976
Switzerland (*n = 17*)	2.5^a^ (0.40–13)			Quinche 1992
Slovenia (*n = 1*)	2.1	0.22	9.5	Byrne et al. 1976
Croatia, eastern (*n = 1*)	1.4			Grgić et al. 1992
Spain, Lugo *(n = 10*)	2.6 ± 1.3 (H)		144	Melgar et al. 2009
	2.3 ± 1.2 (RFB)		115	
Italy, Mt. Amiata; HgS mine (*n = 1*)	23	210	0.11	Bargagli and Baldi 1984
Italy, Reggio Emilia	0.78 (0.42–1.1)^a^			Cocchi et al. 2006
China, Guangdong (*n* = 7)	0.04 (0.01–0.18)			Shen and Yu 2008

*H* hymenophore, *RFB* rest of fruit body

^a^number of fruit bodies, 95 % confidence interval

The contents of Hg in caps and stipes of *C. comatus* in this study correlated positively with the levels of soil contamination (Fig. 2). Earlier studies showed that *C. comatus* has a potential to accumulate mercury in fruiting bodies, e.g., for specimens from the cinnabar (HgS) mining place in Germany with Hg in soil at 83-mg kg^−1^ dm, the content of Hg in two fruiting bodies was at 144-mg kg^−1^ dm, while for cinnabar mining place in Italy with Hg in soil at 210-mg kg^−1^ dm, the content of Hg in a single fruiting body was much less, i.e., at 23-mg kg^−1^ dm. Also high was Hg content in *C. comatus* emerged at the urbanized places in Finland, which showed up to 17-mg kg^−1^ dm (Table 3). In one study, the *C. comatus*, when compared to inorganic Hg has much better potential for bioconcentration of MeHg with a value of BCF reaching 198 (Fischer et al. 1995). Analysis of all sets of data available on Hg in *C. comatus* and soils (Tables 2 and 3) beneath fruiting bodies showed on a positive correlation between degree of soil and mushroom contamination. Hence, *C. comatus* can be considered as a sensitive species and with bioindication potency for soils polluted with Hg.

As mentioned in the introductory section, young fruiting bodies of *C. comatus* are edible and considered excellent if consumed soon after pick-up, while kept in ambient temperature for longer period undergo auto digestion. Given data showed in Tables 2 and 3, the specimens of *C. comatus* emerged in urban soils can be considerably or highly contaminated with mercury. Eating them can provide to a consumer Hg at relatively high dose, while unresolved question is absorption rate of Hg compounds contained in ingested mushroom meal. Mercury is largely retained in flesh of culinary processed (blanched) mushrooms (Falandysz and Drewnowska 2015b), and this phenomenon seems to be more or less a species-specific feature, and if based on a dry matter content, no loss or even a pseudo-enrichment of Hg in a blanched mushrooms could be observed when compared to substrate product (unpublished, JF). Retention of Hg in blanched mushrooms implies on its occurrence in large portion in form of species hardly soluble in water (HgS, HgSe), and this may suggest on limited absorption of Hg contained in a mushroom meal from the alimentary tract.

## References

[CR1] Allen RO, Steinnes E (1978). Concentrations of some potential toxic metals and other trace elements in wild mushrooms from Norway. Chemosphere.

[CR2] Árvay J, Tomáša J, Hauptvogl M, Kopernická M, Kováčik A, Bajčan D, Massányi P (2014). Contamination of wild-grown edible mushrooms by heavy metals in a former mercury-mining area. J Environ Sci Health B.

[CR3] Bargagli R, Baldi F (1984). Mercury and methyl mercury in higher fungi and their relation with substrata in a cinnabar mining area. Chemosphere.

[CR4] Borovička J, Řanda Z, Jelínek E, Kotrba P, Dunn CE (2007). Hyperaccumulation of silver by *Amanita strobiliformis* and related species of the section *Lepidella*. Mycol Res.

[CR5] Borovička J, Kotrba P, Gryndler M, Mihaljevič M, Řanda Z, Rohovec J, Cajthaml T, Stijve T, Dunn CE (2010). Bioaccumulation of silver in ectomycorrhizal and saprobic macrofungi from pristine and polluted areas. Sci Total Environ.

[CR6] Bressa G, Cima L, Costa P (1998). Bioaccumulation of Hg in the mushroom *Pleurotus ostreatus*. Ecotoxicol Environ Saf.

[CR7] Brzostowski A, Falandysz J, Jarzyńska G, Zhang D (2011). Bioconcentration potential of metallic elements by Poison Pax (*Paxillus involutus*) mushroom. J Environ Sci Health A.

[CR8] Brzostowski A, Jarzyńska G, Kojta AK, Wydmańska D, Falandysz J (2011). Variations in metal levels accumulated in Poison Pax (*Paxillus involutus*) mushroom collected at one site over four years. J Environ Sci Health A.

[CR9] Byrne AR, Tušek-Žnidarič M (1990). Studies of the uptake and binding of trace metals in fungi. Part I: accumulation and characterization of mercury and silver in the cultivated mushroom, *Agaricus bisporus*. Appl Organomet Chem.

[CR10] Byrne AR, Ravnik V, Kosta L (1976). Trace element concentrations in higher fungi. Sci Total Environ.

[CR11] Bystrzejewska-Piotrowska G, Pianka D, Bazała MA, Stębrowski R, Manjón JL, Urban PL (2008). Pilot study of bioaccumulation and distribution of cesium, potassium, sodium and calcium in king oyster mushroom (*Pleurotus eryngii*) grown under controlled conditions. Int J Phytoremediation.

[CR12] Cen F, Chen L, Hu Y, Xu H (2012). Chelator-induced bioextraction of heavy metals from artificially contaminated soil by mushroom (*Coprinus comatus*). Chem Ecol.

[CR13] Chojnacka A, Jarzyńska G, Drewnowska M, Nnorom IC, Falandysz J (2012). Yellow-cracking Boletes (*Xerocomus subtomentosus*) mushrooms: content and potential to sequestrate mercury. J Environ Sci Health A.

[CR14] Chudzyński K, Falandysz J (2008). Multivariate analysis of elements content of Larch Bolete (*Suillus grevillei*) mushroom. Chemosphere.

[CR15] Chudzyński K, Bielawski L, Falandysz J (2009). Mercury bio-concentration potential of Larch Bolete, *Suillus grevillei*, mushroom. Bull Environ Contam Toxicol.

[CR16] Cibulka J, Śiśak L, Pulkrab K, Miholova D, Szakova J, Fućikova A, Slamova A, Stehulova I, Barlakova S (1996). Cadmium, lead, mercury and caesium levels in wild mushrooms and forest berries from different localities of the Czech Republic. Sci Agric Biochem.

[CR17] Cocchi L, Vescovi L, Petrini L, Petrini O (2006). Heavy metals in edible mushrooms in Italy. Food Chem.

[CR18] Demirbaş A (2002). Metal ion uptake by mushrooms from natural and artificially enriched soils. Food Chem.

[CR19] Drewnowska M, Falandysz J (2015). Investigation on minerals composition and accumulation by popular edible mushroom Common Chanterelle (*Cantharellus cibarius*). Ecotoxicol Environ Saf.

[CR20] Drewnowska M, Jarzyńska G, Kojta AK, Falandysz J (2012). Mercury in European Blusher, *Amanita rubescens*, mushroom and soil. Bioconcentration potential and intake assessment. J Environ Sci Health B.

[CR21] Drewnowska M, Lipka K, Jarzyńska G, Danisiewicz-Czupryńska D, Falandysz J (2013). Investigation on metallic elements of Fly Agaric, *Amanita muscaria*, fungus and the forest soils from the Mazurian Lakes District of Poland. Fresenius Environ Bull.

[CR22] Drewnowska M, Nnorom IC, Falandysz J (2014). Mercury in the Tawny Grisette, *Amanita vaginata* Fr. and soil below the fruiting bodies. J Environ Sci Health B.

[CR23] Falandysz J (2002). Mercury in mushrooms and soil of the Tarnobrzeska Plain, southeastern Poland. J Environ Sci Health A.

[CR24] Falandysz J, Borovička J (2013). Macro and trace mineral constituents and radionuclides in mushrooms—health benefits and risks. Appl Microbiol Biotechnol.

[CR25] Falandysz J, Chwir A (1997). The concentrations and bioconcentration factors of mercury in mushrooms from the Mierzeja Wiślana sand-bar, Northern Poland. Sci Total Environ.

[CR26] Falandysz J, Danisiewicz D (1995). Bioconcentration factors (BCF) of silver in wild *Agaricus campestris*. Bull Environ Contam Toxicol.

[CR27] Falandysz J, Drewnowska M (2015). Macro and trace elements in Common Chanterelle (*Cantharellus cibarius*) mushroom from the European background areas in Poland: Composition, accumulation, dietary exposure and data review for species. J Environ Sci Health B.

[CR28] Falandysz J, Drewnowska M (2015). Distribution of mercury in *Amanita fulva* (Schaeff.) Secr. mushrooms: accumulation, loss in cooking and dietary intake. Ecotoxicol Environ Saf.

[CR29] Falandysz J, Bona H, Danisiewicz D (1994). Silver uptake by *Agaricus bisporus* from an artificially enriched substrate. Z Lebensm- Unters -Forsch.

[CR30] Falandysz J, Gucia M, Frankowska A, Kawano M, Skwarzec B (2001). Total mercury in wild mushrooms and underlying soil substrate from the city of Umeå and its surroundings, Sweden. Bull Environ Contam Toxicol.

[CR31] Falandysz J, Szymczyk K, Ichihashi H, Bielawski L, Gucia M, Frankowska A, Yamasaki S (2001). ICP/MS and ICP/AES elemental analysis (38 elements) of edible wild mushrooms growing in Poland. Food Addit Contam.

[CR32] Falandysz J, Bielawski L, Kannan K, Gucia M, Lipka K, Brzostowski A (2002). Mercury in wild mushrooms and underlying soil substrate from the great lakes land in Poland. J Environ Monit.

[CR33] Falandysz J, Lipka K, Gucia M, Kawano M, Strumnik K, Kannan K (2002). Accumulation factors of mercury in mushrooms from Zaborski Landscape Park, Poland. Environ Int.

[CR34] Falandysz J, Brzostowski A, Kawano M, Kannan K, Puzyn T, Lipka K (2003). Concentrations of mercury in wild growing higher fungi and underlying substrate near Lake Wdzydze, Poland. Water Air Soil Pollut.

[CR35] Falandysz J, GuciaM BA, Kawano M, Bielawski L, Frankowska A, Wyrzykowska B (2003). Content and bioconcentration of mercury in mushrooms from northern Poland. Food Addit Contam.

[CR36] Falandysz J, Lipka K, Kawano M, Brzostowski A, Dadej M, Jędrusiak A, Puzyn T (2003). Mercury content and its bioconcentration factors at Łukta and Morąg, Northeastern Poland.. J Agric Food Chem.

[CR37] Falandysz J, Frankowska A, Jarzyńska G, Dryżałowska A, Kojta AK, Zhang D (2011). Survey on composition and bioconcentration potential of 12 metallic elements in King Bolete (*Boletus edulis*) mushroom that emerged at 11 spatially distant sites. J Environ Sci Health B.

[CR38] Falandysz J, Kojta AK, Jarzyńska G, Drewnowska A, Dryżałowska A, Wydmańska D, Kowalewska I, Wacko A, Szlosowska M, Kannan K, Szefer P (2012). Mercury in Bay Bolete *Xerocomus badius*: bioconcentration by fungus and assessment of element intake by humans eating fruiting bodies. Food Addit Contam A.

[CR39] Falandysz J, Dryżałowska A, Saba M, Wang J, Zhang D (2014). Mercury in the fairy-ring of *Gymnopus erythropus* (Pers.) and *Marasmius dryophilus* (Bull.) P. Karst. mushrooms from the Gongga Mountain, Eastern Tibetan Plateau. Ecotoxicol Environ Saf.

[CR40] Falandysz J, Krasińska G, Pankavec S, Nnorom IC (2014). Mercury in certain Boletus mushrooms from Poland and Belarus. J Environ Sci Health B.

[CR41] Falandysz J, Zhang J, Wang Y, Krasińska G, Kojta A, Saba M, Shen T, Li T, Liu H (2015). Evaluation of the mercury contamination in mushrooms of genus *Leccinum* from two different regions of the world: accumulation, distribution and probable dietary intake. Sci Total Environ.

[CR42] Falandysz J, Zhang J, Wang Y, Saba M, Krasińska G, Wiejak A, Li T (2015b) Evaluation of the mercury contamination in Fungi *Boletus* species from latosols, lateritic red earths, and red and yellow earths in the Circum-Pacific Mercuriferous Belt of Southwestern China. PLoSONE 10(11):e0143608. doi:10.1371/journal.pone.0143608 10.1371/journal.pone.0143608 PMC4659685 26606425

[CR43] Fischer RG, Rapsomanikis S, Andreae MO, Baldi F (1995). Bioaccumulation of methylmercury and transformation of inorganic mercury by macrofungi. Environ Sci Technol.

[CR44] Frankowska A, Ziółkowska J, Bielawski L, Falandysz J (2010). Profile and bioconcentration of minerals by King Bolete (*Boletes edulis*) from the Płocka Dale in Poland. Food Addit Contam B.

[CR45] Gadd GM, Rhee YJ, Stephenson K, Wie Z (2012). Geomycology: metals, actidines and biominerals. Environ Microbiol Rep.

[CR46] Galanda D, Mátel L, Strišovská J, Dulanská S (2014). Mycoremediation: the study of transfer factor for plutonium and americium uptake from the ground. J Radioanal Nucl Chem.

[CR47] Garciá MÁ, Alonso J, Melgar MJ (2005). *Agaricus macrosporus* as a potential bioremediation agent for substrates contaminated with heavy metals. J Chem Technol Biotechnol.

[CR48] Grgić J, Mandić M, Grgić Z, Manić Z (1992). Živa i arsen u samoniklim jestivim i nejestivim gljivama. Farm Glas.

[CR49] Gucia M, Jarzyńska G, Rafał E, Roszak M, Kojta AK, Osiej I, Falandysz J (2012). Multivariate analysis of mineral constituents of edible Parasol Mushroom (*Macrolepiota procera*) and soils beneath fruiting bodies collected from Northern Poland. Environ Sci Pollut Res.

[CR50] Gucia M, Jarzyńska G, Kojta AK, Falandysz J (2012). Temporal variability in twenty chemical elements content of Parasol Mushroom (*Macrolepiota procera*) collected from two sites over a few years. J Environ Sci Health B.

[CR51] Hu Y, Cheng H (2012). Mercury risk from fluorescent lamps in China: current status and future perspective. Environ Int.

[CR52] Jarzyńska G, Falandysz J (2011). The determination of mercury in mushrooms by CV-AAS and ICP-AES techniques. J Environ Sci Health A.

[CR53] Jarzyńska G, Chojnacka A, Dryżałowska A, Nnorom IC, Falandysz J (2012). Concentrations and bioconcentration factors of minerals by Yellow-cracking Bolete (*Xerocomus subtomentosus*) mushroom collected in Noteć Forest, Poland. J Food Sci.

[CR54] Jiang J, Qin C, Shu X, Chen R, Song H, Li Q, Xu H (2015). Effects of copper on induction of thiol-compounds and antioxidant enzymes by the fruiting body of *Oudemansiella radicata*. Ecotoxicol Environ Saf.

[CR55] Kojo M-R, Lodenius M (1989). Cadmium and mercury in macrofungi—mechanisms of transport and accumulation. Angew Bot.

[CR56] Kojta A, Jarzyńska G, Falandysz J (2012). Mineral composition and heavy metal accumulation capacity of Bay Bolete (*Xerocomus badius*) fruiting bodies collected near a former gold and copper mining area. J Geochem Explor.

[CR57] Kojta AK, Wang Y, Zhang J, Li T, Saba M, Falandysz J (2015). Mercury contamination of fungi genus *Xerocomus* in the Yunnan Province in China and the region of Europe. J Environ Sci Health A.

[CR58] Krasińska G, Falandysz J (2015). Mercury in Hazel Bolete *Leccinum griseum* and soil substratum: distribution, bioconcentration and probable dietary exposure. J Environ Sci Health A.

[CR59] Krasińska G, Falandysz J (2015). Mercury in Orange Birch Bolete *Leccinum versipelle* and soil substratum: Bio-concentration by mushroom and probable dietary intake by consumers. Environ Sci Pollut Res.

[CR60] Kubrová J, Zigovác A, Řanda Z, Rohovec J, Gryndler M, Krausová I, Dunn CE, Kotrba P, Borovička J (2014). On the possible role of macrofungi in the biogeochemical fate of uranium in polluted forest soils. J Hazard Mater.

[CR61] Kuusi T, Laaksovirta K, Liukkonen-Lilja H, Lodenius M, Piepponen S (1981). Lead, cadmium, and mercury contents of fungi in the Helsinki area and in unpolluted control areas. Z Lebensm Unters Forsch.

[CR62] Laaksovirta K, Lodenius M (1979). Mercury content of fungi in Helsinki. Ann Bot Fenn.

[CR63] Lassoe T, Del Conte A, Lincoff G (1996). The mushroom book.

[CR64] Lepp NW, Harrison SCS, Morrell BG (1987). A role for *Amanita muscaria* L. in the circulation of cadmium and vanadium in non-polluted woodland. Environ Geochem Health.

[CR65] Liukkonen-Lilja H, Kuusi T, Laaksovirta K, Lodenius M, Piepponen S (1983). The effect of lead processing works on the lead, cadmium and mercury content of fungi. Z Lebensm- Unters -Forsch.

[CR66] Lodenius M, Kuusi T, Laaksovirta K, Liukkonen-Lilja H, Piepponen S (1981) Lead, cadmium and mercury contents of fungi in Mikkeli, SE Finland. Ann Bot Fennici 18:183–186 10.1007/BF01042578 7303918

[CR67] McGrath SP, Zhao F-J (2003). Phytoextraction of metals and metalloids from contaminated soils. Curr Oppin Biotechnol.

[CR68] Melgar MJ, Alonso J, Garcia MÁ (2009). Mercury in edible mushrooms and soil. Bioconcentration factors and toxicological risk. Sci Total Environ.

[CR69] Mleczek M, Siwulski M, Mikołajczak P, Gasiecka M, Sobieralski K, Szymańczyk M, Goliński P (2015). Content of selected elements in *Boletus badiu*s fruiting bodies growing in extremely polluted wastes. J Environ Sci Health A.

[CR70] Nasr M, Arp P (2011). Hg concentrations and accumulations in fungal fruiting bodies, as influenced by forest soil substrates and moss carpets. Appl Geochem.

[CR71] Nnorom IC, Jarzyńska G, Drewnowska M, Kojta AK, Pankavec S, Falandysz J (2013). Trace elements in sclerotium of *Pleurotus tuber-regium* (Ósu) mushroom—dietary intake and risk in Southeastern Nigeria. J Food Compos Anal.

[CR72] PIG, 2013 PIG. http://www.mapgeochem.pgi.gov.pl/poland/index.html

[CR73] Quinche J (1976). La pollution mercurielle de diverses especes de champignons. Rev Suisse Agric.

[CR74] Quinche JP (1992). Les teneurs en huit éléments traces des carpophores de *Coprinus comatus*. Mycol Helv.

[CR75] Quinche JP, Dvorak V (1975). Le mercure dans des vegetaux et des sols de Suisse romande. Rech Agron Suisse.

[CR76] Rieder SR, Brunner I, Horvat M, Jacobs A, Frey B (2011). Accumulation of mercury and m ethylmercury by mushrooms and earthworms from forest soils. Environ Pollut.

[CR77] Seeger R, Nützel R (1976). Quecksilbergehalt der Pilze. Z Lebensm Unters Forsch.

[CR78] Shen X, Yu S (2008). Analysis and assessment of heavy metal pollution in edible mushrooms from Guangdong (*in Chinese*). Acta Agriculturae Boreali-occidentalis Sinica.

[CR79] Stijve T, Roschnik R (1974). Mercury and methyl mercury content of different species of fungi. Travaux de chimie alimentaire et d’hygiène.

[CR80] Tel G, Çavdar H, Deveci E, Öztürk M, Duru E, Turkoğlub A (2014). Minerals and metals in mushroom species in Anatolia. Food Addit Contam B.

[CR81] Wang D, Chen L, Jing X, Xu H (2012). Chelator- and surfactant-assisted remediation of heavy metal contaminated soil by *Tricholoma lobayense* Heim. Chin J Appl Environ Biol.

[CR82] Wiejak A, Wang Y, Zhang J, Falandysz J (2014). Bioconcentration potential and contamination with mercury of pantropical mushroom *Macrocybe gigantea*. J Environ Sci Health B.

[CR83] Xu J, Garcia Bravo A, Lagerkvist A, Bertilsson S, Sjöblom R, Kumpiene J (2015). Sources and remediation techniques for mercury contaminated soil. Environ Int.

[CR84] Zhang W, Hu Y, Cao Y, Huang F, Xu H (2012). Tolerance of lead by the fruiting body of *Oudemansiella radicata*. Chemosphere.

[CR85] Zhang J, Li T, Yang Y-L, Liu H-G, Wang Y-Z (2015). Arsenic concentrations and associated health risks in *Laccaria* mushrooms from Yunnan (SW China). Biol Trace Elem Res.

